# Topical application of Chlorin e6-PVP (Ce6-PVP) for improved endoscopic detection of neoplastic lesions in a murine colitis-associated cancer model

**DOI:** 10.1038/s41598-020-69570-2

**Published:** 2020-08-04

**Authors:** Ingo Ganzleben, Martin Hohmann, Alexander Grünberg, Jean Gonzales-Menezes, Michael Vieth, Eva Liebing, Claudia Günther, Veronika Thonn, Daniel Beß, Christoph Becker, Michael Schmidt, Markus F. Neurath, Maximilian J. Waldner

**Affiliations:** 1 Department of Medicine 1, University Hospital, Friedrich-Alexander-Universität Erlangen-Nürnberg, Erlangen, Germany; 2 Deutsches Zentrum Immuntherapie (DZI), Erlangen, Germany; 3 Erlangen Graduate School in Advanced Optical Technologies (SAOT), Friedrich-Alexander-Universität Erlangen-Nürnberg, Erlangen, Germany; 4 Institute of Pathology, Klinikum Bayreuth, Bayreuth, Germany

**Keywords:** Colonoscopy, Diagnostic markers, Cancer models, Cancer screening

## Abstract

Screening colonoscopy is crucial in reducing the mortality of colorectal cancer. However, detecting adenomas against the backdrop of an inflamed mucosa (e.g. in ulcerative colitis) remains exceedingly difficult. Therefore, we aimed to improve neoplastic lesion detection by employing a fluorescence-based endoscopic approach. We used the well-established murine AOM/DSS model to induce inflammation-driven carcinogenesis in the colon. In our diagnostic approach, we evaluated Chlorin e6 polyvinylpyrrolidone (Ce6-PVP)-based fluorescence endoscopy in comparison to standard white-light endoscopy. A specialized pathologist then analyzed the histology of the detected lesions. Complementary in vitro studies were performed using human cell lines and a murine organoid system. Ce6-PVP-based fluorescence endoscopy had an improved detection rate of 100% (8/8) in detecting high-grade dysplasias and carcinomas over white-light detection alone with 75% (6/8). Trade-off for this superior detection rate was an increased rate of false positive lesions with an increase in the false discovery rate from 45% for white-light endoscopy to 81% for fluorescence endoscopy. We demonstrate in a proof-of-concept study that Ce6-PVP-based fluorescence endoscopy is a highly sensitive red flag technology to identify biopsy-worthy lesions in the colon.

## Introduction

Achieving high adenoma detection rates during screening colonoscopy remains a formidable challenge as small or flat lesions are particularly difficult to detect^1–3^. Among the technologies used to improve white-light endoscopy and increase detection rates, fluorescence-enhanced endoscopy has proven exceptionally promising. Specifically targeted fluorophores have been applied both systemically (intravenously) and topically (e.g. via enema)^4, 5^ to detect dysplastic lesions in the colon with overall encouraging results.

However, how to best identify relevant sites for biopsy in inflamed colonic mucosa, e.g. in inflammatory bowel diseases such as ulcerative colitis, remains an open question. It is generally accepted that the disease-specific risk for developing a colorectal carcinoma begins roughly 7 years after diagnosis of UC and a recent meta-analysis has supported that the risk for CRC is positively correlated with inflammatory activity^6^. However, discerning non-neoplastic inflammatory alterations from neoplastic lesions during colonoscopy gets increasingly difficult with increasing inflammatory activity. Conflicting data on the best approach for surveillance strategies necessitate further research into emerging technologies such as fluorescence endoscopy.

To address this challenge, we developed a fluorescence endoscopy approach employing the established substance Chlorin e6-polyvinylpyrrolidone (Ce6-PVP), which significantly improves on the existing specificity for malignant tissue displayed by Ce6 or other derivatives such as Ce6-DMSO^7^, as a diagnostic fluorophore.

The chemical modification of coupling Chlorin e6 to a polyvinylpyrrolidone (PVP) polymer in a mass ratio of 1:1 yields the high molecular weight compound Ce6-PVP (12,000 g/mol), which is commercially available as “Photolon” (Belmedpreparaty, Belarus). In this context, reduced aggregate formation in aqueous solutions^8^ and increased cellular uptake over the plasma membrane^9^ compared to Ce6 are relevant advantages of Ce6-PVP.

Previous studies have so far shown promising results regarding the general use of Ce6-PVP in diagnostic fluorescence imaging in vivo using xenograft models of bladder tumors^10^ or nasopharynx carcinomas^11^.

In our current study, we report for the first time on the topical application of Ce6-PVP during colonoscopy in an inflammation-driven murine tumor model to successfully achieve highly sensitive detection of colonic neoplasms and areas of interest.

## Results

### Ce6-PVP is suitable for optical imaging of colorectal cancer cells in vitro

**Figure 1:**
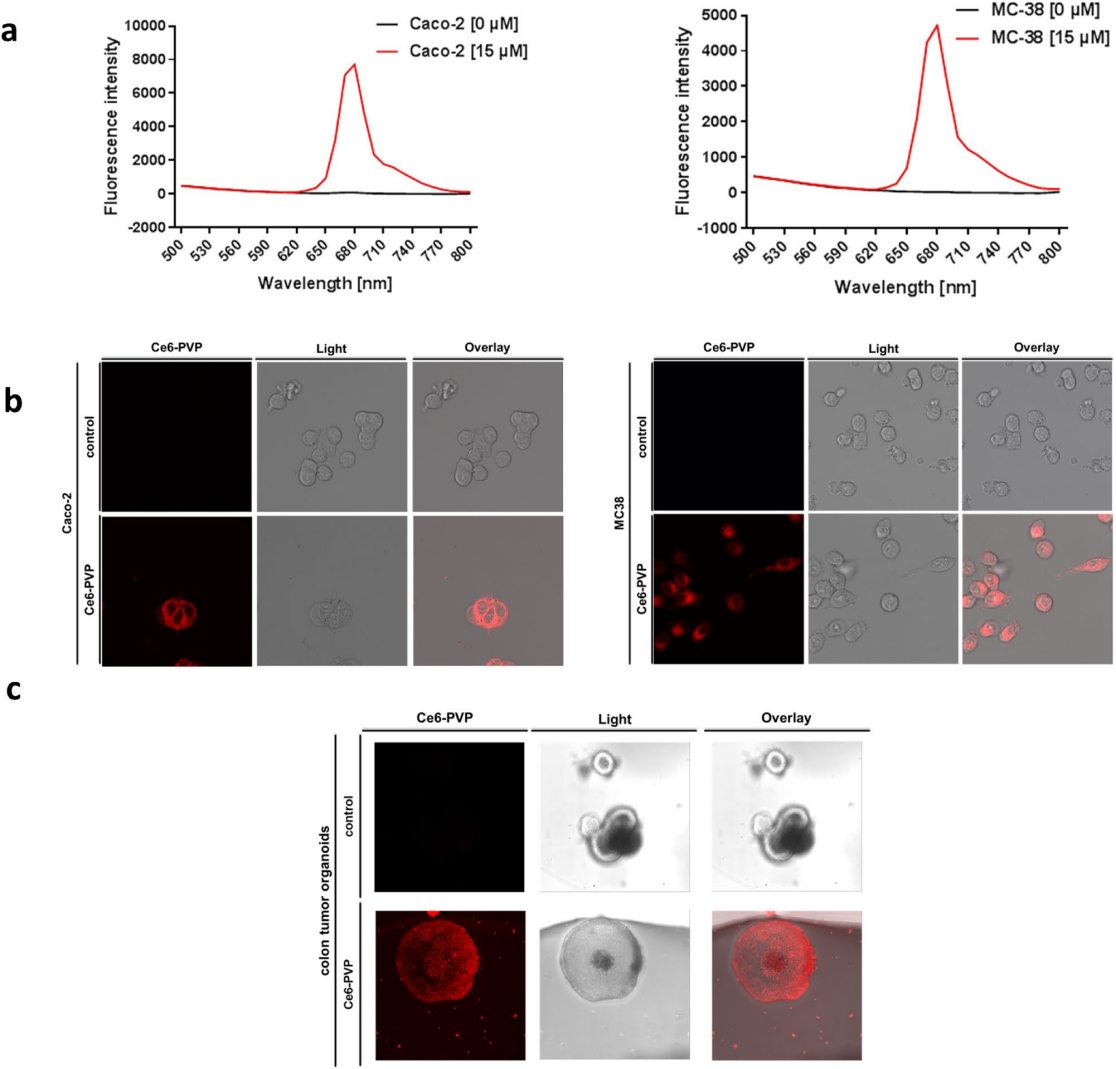
Ce6-PVP is suitable for optical imaging of colorectal cancer cells in vitro*.* (**a**, **b**) MC-38 and Caco-2 cells were either treated with Ce6-PVP [15 µM] for 20 min or left untreated as controls. Cells were washed afterwards with PBS to remove unbound Ce6-PVP. (**a**) Emission scan of Ce6-PVP fluorescence intensity (excitation wavelength 405 nm) with an optical photometer 4 h after treatment. X-axis: Emission wavelength [nm], Y-axis: fluorescence intensity. Shown is one representative measurement out of three (Caco-2) or two (MC38) iterations of the experiment. (**b**) Confocal microscope images. Imaging sequence was begun within 1 h after treatment. Images are representative of two independent experiments (Caco-2) or display one experiment (MC38). (**c**) Murine colon tumor organoids were generated and either treated with Ce6-PVP [15 µM] for 30 min or left untreated as controls. Organoids were washed with PBS to remove unbound Ce6-PVP and imaged afterwards. Confocal microscope images. Imaging sequence was begun within 1 h after treatment. Images are representative of two independent experiments.

In a first step, we evaluated Ce6-PVP for the optical detection of human (Caco-2) and murine (MC38) colorectal cancer cells in vitro (Fig. 1). In both cell types, a relevant fluorescence signal was detectable by fluorescence intensity measurements (Fig. 1a) and by visualization of stained cells by confocal microscopy (Fig. 1b).

In a next step, we transferred our findings into a more physiological model. Accordingly, we generated organoids from murine colon tumors and again performed fluorescence signal detection by confocal microscopy after briefly exposing the organoids to Ce6-PVP (Fig. 1c). As a result, we found that colon tumor organoids emitted a reliably strong Ce6-PVP signal suitable for detection.

### Ce6-PVP is suitable for endoscopic imaging of colorectal neoplasias in vivo

**Figure 2:**
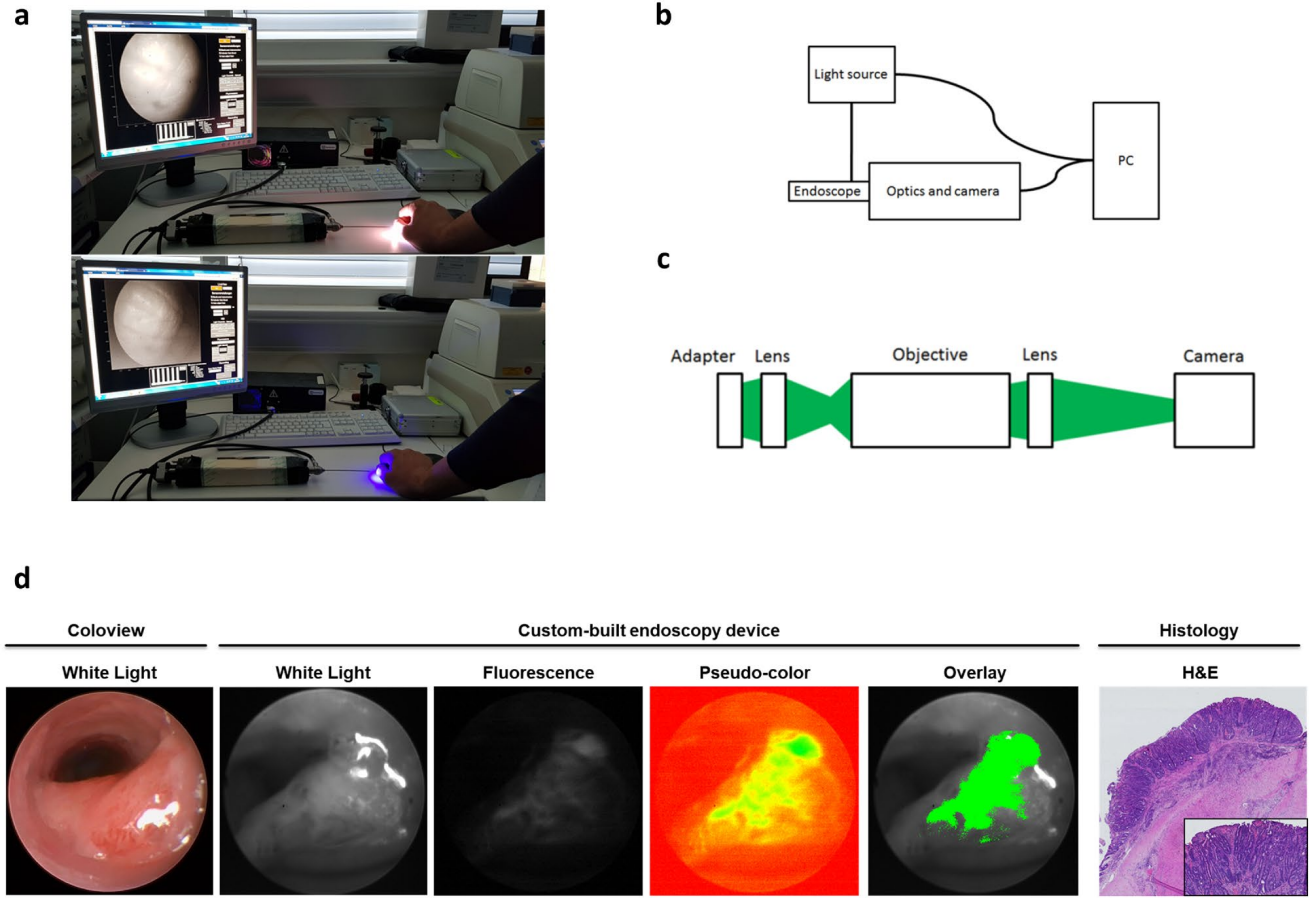
Ce6-PVP is suitable for endoscopic imaging of colorectal neoplasias in vivo*. *(**a**) Visual display of the custom-built endoscopy device hardware and its software interface in white-light mode (upper panel) and fluorescence mode (lower panel) using the example of human tissue. (**b, c**) Schematic drawings of (**b**) the endoscopy system and (**c**) the optics unit setup. A detailed description of the systems can be found in the “Material and Methods” section. (**d**) Representative images displaying a murine colorectal tumor in different imaging modalities. The white-light image of the coloview system (left) represents the current standard technology for murine colonoscopy. Our custom-built endoscopy device (middle) is capable of alternatingly imaging a white-light signal and a Ce6-PVP-based fluorescence signal during the same endoscopy session. The fluorescence image was post-processed by our algorithm and afterwards displayed as an overlay on the white-light image. H&E staining of the tissue displays the neoplastic character of the lesion. An experienced pathologist confirmed the diagnosis of a carcinoma based on 3 µm sections of a swiss roll preparation of the colon at variable intervals. Localization relative to the anocutaneous line was used to assure correct matching of histological and endoscopic lesions.

To expand on our initial in vitro data, we next conceptualized an endoscopic imaging device to allow for Ce6-PVP fluorescence detection in vivo (Fig. 2a). We designed a novel endoscopy device with a custom-built optics unit operated by a control and data processing software written in Matlab (Fig. 2b). The optics unit (Fig. 2c) was built with a particular emphasis on modifiability of this prototype in mind.

We employed an established model of inflammation-driven carcinogenesis (AOM/DSS) to test our newly developed system in a situation comparable to the conditions found in human patients with long-standing inflammatory bowel disease.

We tested our device on a colonic lesion macroscopically classified as “definitive carcinoma” based on white-light coloview system endoscopy (Fig. 2d left picture). After topical application of Ce6-PVP, our system was capable of detecting the fluorescence signal in addition to the standard white-light image with the push of a button. Beyond that, our data processing routine was able to optimize the fluorescence image for display and create an overlay image of the fluorescence and white-light images (Fig. 2d middle column). The diagnosis of carcinoma was histologically confirmed by a specialized pathologist (Fig. 2d right column).

### Ce6-PVP-based fluorescence endoscopy improves sensitivity for neoplastic lesions and helps to identify biopsy-worthy areas during colonoscopy in vivo

After this successful proof-of-concept, we evaluated whether our fluorescence-based detection system could support an endoscopist in detecting neoplasias and biopsy-worthy regions in the context of inflammatory carcinogenesis.

**Figure 3:**
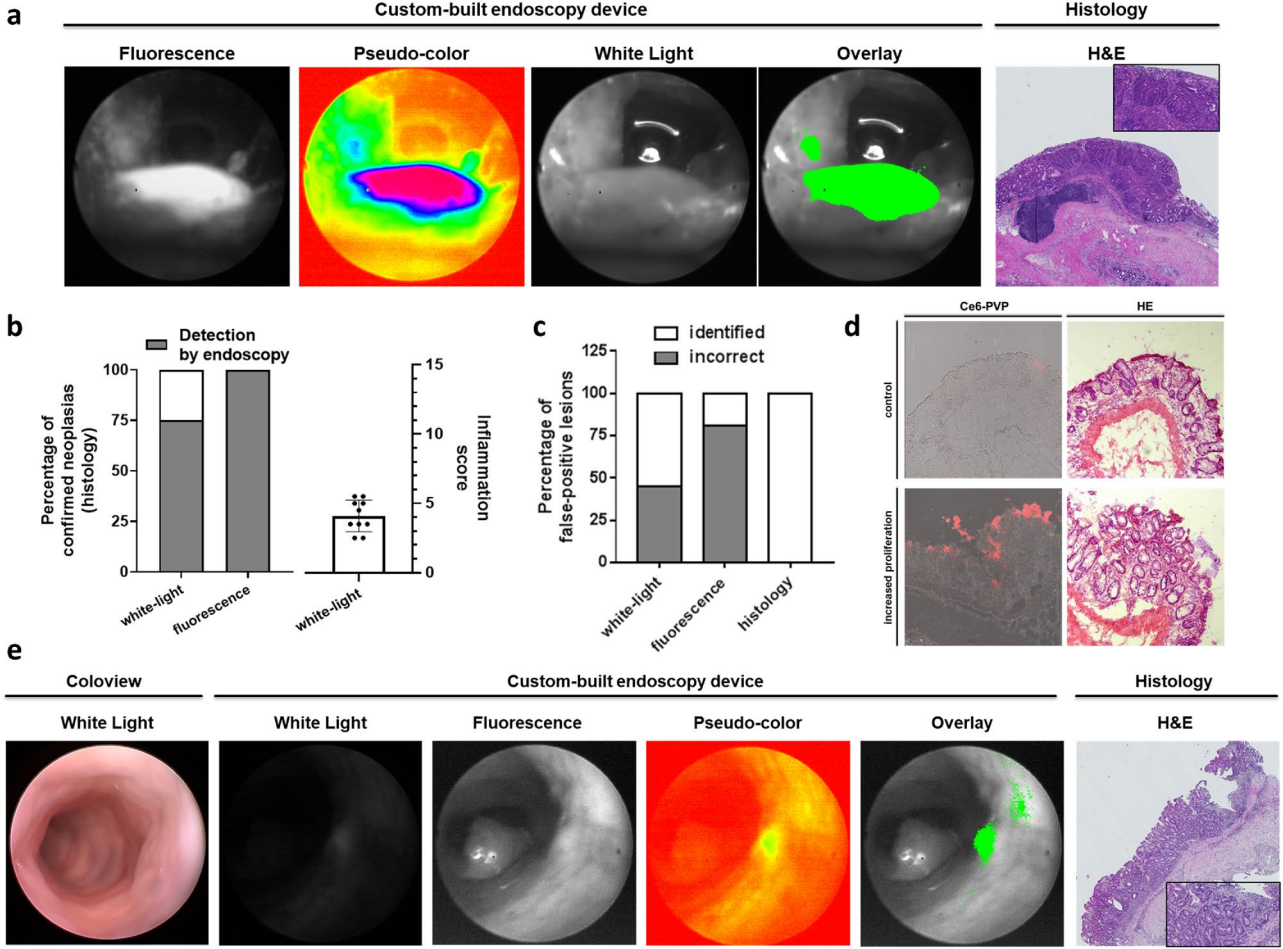
Ce6-PVP-based fluorescence endoscopy improves the detection rate of neoplastic lesions and helps to identify biopsy-worthy areas during colonoscopy in vivo*.* (**a**) Representative images displaying an easy-to-miss murine colorectal lesion localized near the anocutaneous line in different imaging modalities. The tumor was missed using white-light endoscopy. Due to its intense fluorescence signal, the tumor was directly detected during fluorescence endoscopy. The post-processed fluorescence signal and the fluorescence/white-light overlay illustrate improved discriminability of the tumor against its surroundings compared to white-light endoscopy alone. H&E staining displays the neoplastic character of the lesion. An experienced pathologist confirmed the diagnosis of high-grade dysplasia based on 3 µm sections of a swiss roll preparation of the colon at variable intervals. Localization relative to the anocutaneous line was used to assure correct matching of histological and endoscopic lesions. (**b**) Left diagram: Stacked bar chart displaying the percentage of correctly identified neoplastic lesions. The total amount of histologically proven neoplastic lesions is defined as 100% (n = 8). The detection rate is given for white-light endoscopy (75%, 6/8; coloview) and fluorescence endoscopy (100%, 8/8; custom-built device). Right diagram: Scattered dot plot displaying the endoscopic colonic inflammation score [0 to 15]. Bar chart indicates mean (+ /- standard deviation (SD)). (**c**) Stacked bar chart displaying incorrectly identified lesions as percentage of all identified lesions for white-light, fluorescence, and histology (gold standard test). For white-light endoscopy 45% (5/11 identified lesions) were incorrect in comparison to 81% (34/42 identified lesions) for the fluorescence endoscopy. (**d**) Native confocal microscopy of Ce6-PVP fluorescence (left) and H&E staining (right) of inflamed colonic mucosa (lower panel) compared to unaltered mucosa (upper panel). An experienced pathologist diagnosed increased proliferation due to tissue regeneration (“reactive focus”) based on representative images of stained 7 µm cryo-sections from a colon segment. (**e**) Representative images of a false positive fluorescence lesion in different imaging modalities. Consistent with the findings of Fig. 3d, the fluorescence signal identifies a region with strongly altered epithelium. H&E staining displays the irregular character of the lesion. An experienced pathologist diagnosed increased proliferation based on 3 µm sections at intervals of 20 µm from a colon segment sample. Relative topography of the endoscopically detected lesion to the anal canal was used to match the correct colon segment during collection.

We found that our approach was able to detect neoplastic lesions with an improved detection rate. One of the lesions that remained undetected during white-light endoscopy, for example, was particularly close to the anocutaneous line and located directly on a mucosal fold. It was overlooked in the white-light endoscopy but spotted using our new approach as its strong fluorescence signal guided the attention of the endoscopist (Fig. 3a).

Overall, Ce6-PVP-based fluorescence endoscopy was able to correctly indicate 100% (8/8) of histologically diagnosed high-grade dysplasias and carcinomas including the one displayed in Fig. 2d. In contrast, a skilled endoscopist was only able to correctly discover 75% (6/8) of those lesions (Fig. 3b). Importantly, this improved detection rate was achieved against the backdrop of an inflamed colonic mucosa. The extent of inflammation was assessed during white-light endoscopy on a previously defined scale from 0 to 15^12^. The mean score value was 4.1 with a standard deviation of 1.15, reflecting mild but consistent inflammation in our cohort (Fig. 3b). Trade-off for the improved detection was an increased rate of false positive lesions in our approach compared to white-light endoscopy alone (Fig. 3c). In detail, an experienced researcher identified a total of 11 malignancy suspect lesions during white-light endoscopy of which 6 were histologically confirmed and 5 were false positive resulting in a false discovery rate (FDR) of 45% with a positive predictive value (PPV) of 55%. In comparison, our fluorescence-based approach identified 42 suspect lesions of which 8 were histologically confirmed and 34 were false positives resulting in a false discovery rate (FDR) of 81% and a positive predictive value (PPV) of 19% (Table 1). In order to set this finding into context, we imaged Ce6-PVP on native tissue sections from inflamed colon and found that Ce6-PVP accumulated in regions in which a specialized pathologist diagnosed regenerative foci (Fig. 3d).

**Table 1:** Statistical parameters and detailed endoscopic findings.

	White-light endoscopy	Ce6-PVP-based endoscopy		
Detection rate	75% (6/8)	100% (8/8)		
Positive predictive value	55% (6/11)	19% (8/42)		
False discovery rate	45% (5/11)	81% (34/42)		

	White-Light	Fluorescence	Histology	Histopathological diagnosis
Mouse 1	1	3	0	
Mouse 2	0	2	0	
Mouse 3	0	3	0	
Mouse 4	0	1	0	
Mouse 5	1	2	0	
Mouse 6	1	1	0	
Mouse 7	2	5	2	2 Carcinomas
Mouse 8	4	11	3	3 High-grade dysplasias
Mouse 9	2	8	2	2 High-grade dysplasias
Mouse 10	0	6	1	1 High-grade dysplasia
Total	11	42	8	

Upper part: Cumulative statistical analysis of all detected lesions per modality (white-light or Ce6-PVP-based endoscopy) based on histologically confirmed lesions. Lower part: Detailed overview of detected lesions in each modality per mouse including the histopathological diagnosis and summary of the total number of lesions detected in each modality.

In a next step, we then had a closer look at some of the Ce6-PVP fluorescence positive lesions, which were neither malignant nor dysplastic. Interestingly, we found that some specimens with false positive lesions appeared to display circumscribed areas of highly active cell proliferation such as reactive foci partly with polypoid macroscopic appearance leading to difficulties in distinguishing them from high-grade dysplasias (Fig. 3e).

## Discussion

In summary, our data for the first time demonstrate the feasibility of improving endoscopic detection rates of neoplastic and dysplastic lesions in a murine colitis-associated cancer model by employing fluorescence endoscopy using topically applied Chlorin e6-polyvinylpyrrolidone (Ce6-PVP) as fluorophore.

Fluorescence-enhanced endoscopy represents a particularly promising approach to improve the detection of neoplastic colorectal lesions. Recent studies have for example employed fluorophores coupled with small peptide molecules for the endoscopic detection of sporadic colorectal neoplasias^4, 5, 13^. These peptides had either been identified using a phage display technique^5^ or specifically designed to bind to a known target structure such as c-Met^4^ or the V600E mutation in BRAF^13^. However, a disadvantage of this approach is that detection is limited to lesions carrying the target structure which might only be a particular subset of lesions, e.g. sessile serrated adenomas (SSAs)^13^. This, in turn, necessitates a complex combination of markers in order to assure reliable detection of all relevant lesions. Furthermore, the biological functions of phage display identified targets are most of the time not entirely known yet. Fewer studies exist on targeted approaches focusing on inflammatory carcinogenesis, e.g. in the AOM/DSS model, but generally face the same difficulties^14^.

An approach using a topically applied fluorophore, hexaminolevulinate (HAL), a derivate of 5-aminolevulinic acid (5-ALA), which specifically accumulates in dysplastic lesions, has yielded promising results in sporadic carcinomas^15^ but data regarding inflammatory carcinogenesis are currently lacking. The substance 5-aminolevulinic acid (5-ALA) itself has been evaluated in patients with ulcerative colitis^16^. However, the study excluded patients with active inflammation. Yet, patients with persisting inflammation despite best therapeutic efforts are especially at risk of developing malignancies. Our current study therefore focused on a model in which inflammation, albeit mild, was consistently detectable both endoscopically and histologically.

So far, Ce6 and its derivatives have been studied mainly as prospective agents for the photodynamic therapy of colorectal cancer^17–19^. In this context, preclinical imaging has been performed using the IVIS Spectrum system to detect the fluorescence signal of systemically applied Ce6 polyvinyl alcohol^19^, complementing previous studies visualizing Ce6-PVP using a fluorescence endoscopy system on the accessible surface of a chick chorioallantoic membrane in a bladder cancer model^10^ and on xenografts of a nasopharynx carcinoma^11^.

However, our study with the described endoscope prototype is to the best of our knowledge the first proof-of-concept that Ce6-PVP can be used to image neoplastic lesions in the colon after topical application during live endoscopy.

Limitations of our study are the relatively small cohort size and the question of transferability of our murine data to the human system. Follow-up human studies would also have to incorporate the increasingly available high-definition white-light endoscopy. Furthermore, despite its intended role as a screening test with a focus on successfully improving the detection rate during endoscopy, the relatively high false discovery rate (FDR) and the corresponding relatively low positive predictive value (PPV) of our method remain an obstacle for its introduction into clinical practice. The false discovery rate (FDR) of our experienced endoscopist assessing white-light endoscopy footage was relatively high (45%) based on the inherently difficult task to discern inflammatory alterations from dysplastic lesions. While crucially improving the detection of neoplastic lesions, our fluorescence method also increased the false discovery rate (FDR) to about 81% with a corresponding inverse effect on the positive predictive value (55% vs. 19%). Further refinement of our method by analyzing a larger cohort and establishing additional criteria, such as quantitative cut-off values for fluorescence density and/or the definition of specific fluorescence patterns in follow-up studies, might help to overcome these limitations. A following step will then also be to perform a head-to-head comparison of Ce6-PVP and other established fluorophores, such as HAL, in the setting of inflammatory carcinogenesis with our system. Importantly, tissue alterations by Ce6-PVP were not observed and are also not expected based on our technical setup. Regarding the safety profile, Ce6-PVP is already commercially available as a medical drug under the trademark “Photolon” and has been used in clinical studies for the photodynamic treatment (PDT) of advanced malignancies without relevant systemic toxicity^20, 21^. The vendor (Belmedpreparaty) suggests a dose of about 3 mg/kg body weight with systemic application in combination with an adapted dose of 100 to 600 J/cm^2^ of light exposition (https://www.belmedpreparaty.com/eng/photolon.php). Importantly, murine in vivo data using intravenous application of Ce6-PVP have supported that significant toxicity is dependent on high drug doses (5 mg/kg body weight) and high light doses (200 J/cm^2^) in a narrow drug-light-interval of about 1 h^22^. As there is no natural postinterventional light exposition in the colon, light exposition during the diagnostic procedure would be the only possible concern of direct phototoxicity. However, light exposition intensity in our approach is relatively low so that tissue impairment was not observed in our study and is not to be expected in the human system.

Furthermore, as our approach is based on the topical application of Ce6-PVP in contrast to a systemic intravenous application, relevant systemic side effects including skin photosensitivity are not to be primarily expected. However, initial clinical trials applying Ce6-PVP topically in humans would have to include pharmacokinetic studies monitoring any potential systemic uptake as well as assessment of any adverse events as previously established^15^.

However, even in the unlikely case of relevant potential systemic absorption, Ce6-PVP has significantly improved systemic clearance especially from the skin compartment in murine in vivo experiments compared to Ce6^22^, reducing the time window in which global photosensitivity could occur.

Our study not only demonstrates that Ce6-PVP is capable of effectively labeling high-grade dysplasias and carcinomas, but also that it improves on the sensitivity of white-light endoscopy reaching a detection rate of 100% in our current study by assisting the endoscopist to become aware of easy-to-miss lesions. Trade-off for this improved detection rate was an increased number of false positive lesions. We found, however, that some histological samples with false positive lesions displayed circumscribed regions of increased proliferation making them regions of interest for biopsy. This observation offers another rationale to further evaluate Ce6-PVP-based endoscopy as it might not only improve detection of neoplastic lesions but may also guide the endoscopist to biopsy-worthy regions improving on the current standard of random biopsies during SD white-light endoscopy.

In conclusion, Ce6-PVP-based endoscopic fluorescence imaging is an approach that might have the potential to assist clinicians in detecting otherwise difficult to detect dysplastic and neoplastic lesions during screening colonoscopies in patients with underlying inflammatory bowel disease in the future. However, the high false discovery rate needs to be addressed first e.g. in follow-up studies by establishing quantitative cut-offs.

## Methods

### Cell culture and Chlorin e6-PVP (Ce6-PVP) treatment in vitro

The mouse colorectal adenocarcinoma cell line MC38 and the human colorectal adenocarcinoma cell line Caco-2 were maintained in D10 medium which was prepared by using Dulbecco's Modified Eagle Medium (41966029, Thermo Fisher Scientific) supplemented with 10% (v/v) Fetal Bovine Serum (FBS, 10270-106, Gibco/Thermo Fisher Scientific) and 1% (v/v) Penicillin–Streptomycin (P4333, Sigma-Aldrich).

For the experiments, cells were incubated with Ce6-PVP [15 µM] in a cell culture incubator at standard conditions (37 °C, 5% CO_2_) for 20 min. Cells were then washed repeatedly with Dulbecco’s Phosphate Buffered Saline Modified (D8537, Sigma-Aldrich) and again kept in D10 medium until read-out.

An optical photometer (Infinite 200 Pro, Tecan Group) was used to perform an emission scan (420–840 nm) with excitation at a wavelength of 405 nm. The photometer read-out was performed in PBS in a 96-well plate. For confocal microscopy, cells were grown in glass chamber slides (80826, ibidi) and kept in colorless (phenol-red free) DMEM/HamF12 medium (D6434, Sigma) with 10% FBS during the measurement to allow for a prolonged measurement time.

### Organoid culture and Chlorin e6-PVP (Ce6-PVP) treatment in vitro

Murine tumor organoids were generated based on the APCmin/+ model and isolated as described previously^23^ with minor modifications. In brief, tumors were isolated and incubated with 2 mM EDTA on ice. Tumor cells were then detached from the mesenchyme by incubation with a digestion buffer (TrypLE (1x), 2,5% FCS, Pen/Strep Amphotericin Mix 1x, Type IV Collagenase (0.025 g/50 ml), and Type II dispase (0.00625 g/50 ml) for 2 h at 37 °C. Afterwards, the sample was shaken briefly and the supernatant was passed through a 70 µm strainer and collected into a 50 ml tube. Then, centrifugation was performed at 200 g followed by an additional washing step both at 4 °C for 5 min. Tumor cells were then plated into matrigel and cultured in basal culture medium (BCM) consisting of DMEM/F12, HEPES (10 nM), GlutaMax (2 mM), and Pen/Strep Amphotericin Mix (1x). BCM was completed to complete culture medium (CCM) by adding B27 supplement (1x), Acetylcysteine (1 mM), and EGF (50 ng/ml).

For the experiments, organoids were incubated with Ce6-PVP [15 µM] in a cell culture incubator at standard conditions (37 °C, 5% CO_2_) for 30 min. Organoids were then washed repeatedly with Dulbecco’s Phosphate Buffered Saline Modified (D8537, Sigma-Aldrich) and kept in colorless (phenol-red free) DMEM/HamF12 medium (D6434, Sigma) with 10% FBS during the measurement to allow for a prolonged measurement time.

### Colitis-associated cancer (CAC) mouse model

C57BL/6 mice were acquired from the animal facility of the University of Erlangen-Nürnberg. Inflammation-driven tumorigenesis was induced as previously described^24^. In brief, mice were injected intraperitoneally with a single dose of the mutagenic agent Azoxymethane (AOM), followed by three intermittent one-week-cycles of dextran sodium sulfate (DSS) in the drinking water. A total of n = 10 mice were used and analyzed after the third DSS cycle. Animal studies were approved by the Institutional Animal Care and Use Committee of the University of Erlangen-Nürnberg and the government of Lower Franconia. All methods were performed in accordance with the relevant guidelines and regulations.

### Endoscopy-based white-light and fluorescence imaging after Ce6-PVP treatment in vivo

For all endoscopic procedures, mice were anesthetized using isoflurane and the colon was flushed with tap water right before the procedure to clean the bowel. Air was carefully insufflated into the colon to allow for full visualization of the distended bowel wall. Based on the technical specifications of the rigid telescope unit of the Coloview system which was used, imaging was possible up to 4 cm into the colon for both white-light and fluorescence endoscopy depending on the individual anatomical conditions^25^.

White-light endoscopy was performed using the Coloview high-resolution mouse endoscopy system as described previously^12, 26^. The obtained images and videos were archived for later systematic blinded assessment by an experienced researcher based on an adapted version of the inflammation and tumor scoring system previously described by Becker and colleagues ranging from 0 to 15 points in total^12^.

In order to achieve an unbiased interpretation of the detected fluorescence signals by the investigators, conventional white-light endoscopy and fluorescence imaging were not performed during the same measurement session but with a latency of few days. Mice were prepared for colonoscopy and Ce6-PVP [1 mg/ml] was then applied topically to the colon. Application was performed via a custom-built applicator system over the standard endoscopic working channel during withdrawal of the endoscope. Fluorescence in vivo imaging was performed after an incubation period of about 8 min using the prototype of a newly developed endoscopy device for sequential fluorescence and white-light imaging with a filter set optimized for detection of the Ce6 signal. The definition for positivity of the Ce6-PVP-based fluorescence was based on a qualitative cut-off: regions with a sufficient and clearly distinguishable, stronger signal compared to the background of the fluorescence signal were considered positive. No additional quantitative cut-off was applied. Assessment was performed by two authors with experience in endoscopy as well as fluorescence-based imaging in real-time during the endoscopy. Images and videos were acquired electronically for later detailed analysis. The mice were sacrificed at the end of the fluorescence endoscopy session and tissue samples were collected for pathological analysis as described in the “histology” section.

### Equipment and settings

Confocal microscopy was performed using the Leica TCS SP5 II confocal microscope system. H&E slides were either digitalized using the Hamamatsu NanoZoomer (C9600-12) with the NDP.scan software or in some cases imaged with the Leica DMI4000 B microscope system with the Leica DFC420C color camera for bright-field microscopy.

Images of scanned slides were analyzed using the NDP.view 2 software. Images of microscopy samples were globally adjusted for contrast to correctly represent the stained original slide.

### Technical specifications of the endoscopy system

Fluorescence imaging was conducted with a newly developed mouse endoscopy system based on the Coloview System by Karl Storz. As depicted in Fig. 2, the new system utilized only the fiber-based telescope unit of the Coloview system. The telescope unit was connected to an external light source (Lumencor spectra 7-LCR-XA, Beaverton) by means of a liquid light guide and to the endoscope head and a unit consisting of the optics and the camera. During endoscopy, the optics part images the incoming light signal onto the camera. The overall system is controlled by a self-written Matlab (The MathWorks, Inc.) program running on a customary in the trade personal computer hardware. The Matlab program in particular controls the external light source, the input and output of the primary data and creates the graphical user interface (GUI) that faces the end user. To facilitate coordination of these aforementioned components, the video signal serves as a master clock for e.g. the external light source.

The optics unit incorporates two achromatic lenses with an apochromatic objective in between. The signal initially travels from the endoscopy telescope unit via an adaptor to the first lens which has a focal length of 35 mm. This lens then conveys the real image to a 10X infinity corrected plan apochromat objective (Mitutoyo). The objective, which has a numerical aperture (NA) of 0.28 and a working distance of 34 mm, images the received data set to infinity. Using a 100 mm achromatic second lens, the image is finally transmitted from infinity to the camera unit (Basler ace ac2000-165ac USB3 color, Basler AG).

Our design choices regarding the optics unit were guided by the two main goals to prevent chromatic aberrations and to create an easily modifiable system for later expansion.

We were able to reduce chromatic aberrations to minor occurrences in the outer parts of the images by using a 10X infinity corrected plan apochromat objective in combination with the achromatic lenses. Regarding later adaptability, we used an infinity corrected objective permitting integration of a second camera with minor changes to the optical system such as inserting a chromatic beam-splitter in between the objective and the second lens without altering the beam path.

Overall, the set-up is distinguished by the nearly complete absence of barrel distortion. Only small distortions are found.

### Histology

In brief, no biopsies were taken during the endoscopy itself due to technical limitations. Instead, locations of lesions were protocolled during the fluorescence endoscopy defined by their relative distance to the anus. Mice were then sacrificed directly after fluorescence endoscopy and histological samples were systematically assessed. Exact matching of histologically proven lesions to endoscopically observed ones was assured by either cutting the colon sample into predefined segments numbered relative to their position to the anus and cutting and analyzing continuous 3 µm sections of these entire specimens or by using the swiss roll technique which allowed directly measuring the distance of a lesion from the anus. In detail, colon samples were formalin-fixed and paraffin-embedded, cut into 3 µm thin histological sections, and stained according to Mayer’s hematoxylin & eosin protocol. Histological diagnosis was performed by a specialized pathologist. Inflammation scoring was performed on a scale from 0 to 10 points as described previously^27^. Colonic tissue was either embedded in form of precut segments of the colon or using the “swiss roll” technique in which the gut was cut open longitudinally and rolled outside first on a wooden toothpick.

In order to perform native imaging of Ce6-PVP in colonic tissue using a confocal microscope (Fig. 3d), colon tissue was snap frozen and embedded in Tissue-Tek (4583, Sakura) directly after explantation. Samples were then cut into 7 µm thin sections, imaged, and afterwards stained according to Mayer’s hematoxylin & eosin protocol.

### Reagents

Azoxymethane (AOM) was purchased from Sigma-Aldrich (St. Louis, United States). Dextran sulfate sodium salt (DSS) was purchased from MP Biomedicals (Santa Ana, California, USA).

Chlorin e6-polyvinylpyrrolidone was purchased from Belmedpreparaty (Belarus) and was dissolved in sterile Dulbecco’s Phosphate Buffered Saline Modified (DPBS) (D8537, Sigma-Aldrich) in a concentration of 1 mg/ml (80 µM), which was used in all in vivo experiments. For the in vitro experiments, the Ce6-PVP stock solution was diluted in culture medium directly before usage to achieve a final concentration of 200 µg/ml (15 µM).

## Data Availability

The datasets generated and analysed during the current study are available from the corresponding author on reasonable request.
